# Comparison of the English and French versions of the CASPer® Test in a bilingual population

**DOI:** 10.15694/mep.2018.0000281.1

**Published:** 2018-12-11

**Authors:** Christopher Zou, Meghan McConnell, John Leddy, Patrick Antonacci, Geneviève Lemay

**Affiliations:** 1 Altus Assessments; 2 University of Ottawa

**Keywords:** Assessment, Admissions, Evaluation, Psychometrics, Validity, CASPer, French, Language

## Abstract

This article was migrated. The article was marked as recommended.

Objective

The University of Ottawa MD program has two different streams to which candidates may apply: a francophone stream and an anglophone stream. As the admissions office receives applications in both French and English, they are required to ensure that the tools used to assess candidates are psychometrically equivalent across both streams. CASPer is a standardized test they recently adopted to assess the non-cognitive competencies of applicants and is offered in both English and French. The objective of this study is to compare the psychometric properties of the English and French versions of CASPer.

Methods

We collected data from all CASPer test-takers across three cohorts (n = 12,463; entry 2016, entry 2017, entry 2018). We first compared the difficulty of the test between the French and English version using proxy indicators (i.e., time to completion, typing speed). We then compared the psychometric properties of the two versions based on their internal-consistency reliability and applicant acceptability.

Results

There were some indications that the French version may be slightly more difficult than the English version of the CASPer test. However, it is unclear whether this difficulty is due to the difficulty of the individual test items or to differences in the characteristics of the cohort. Nevertheless, a comparison of the psychometric indicators suggests that both French and English versions of CASPer are psychometrically sound and equivalent.

Conclusion

Although CASPer scores cannot be directly compared between the English and French versions, the psychometric properties of the assessment were retained across the two versions. These results provide preliminary evidence that the psychometric strengths of the English version of CASPer likely extend to the French version of the assessment.

## Introduction

There are a number of bilingual medical programs worldwide that train physicians to serve patients in multiple languages. One of the major challenges of bilingual institutions is making the admissions process fair and equivalent across languages. Doing so ensures that all applicants meet the required standards and have the desired competencies for entry into their respective programs or language streams. However, many standardized tests, such as the MCAT, are only offered in one language. This severely limits the number of available assessment tools to guide admissions decisions.

Canada is a bilingual country with two official languages: French and English. Although the majority of Canadians’ first language is English, 23.4% of Canadians identify French as the language spoken at home ( Statistics Canada, 2016). Although the French-speaking population resides primarily in Quebec, where 50.0% of residents do not speak English, a significant number of Canadians identify as francophones in the neighbouring provinces of Ontario and New Brunswick ( Statistics Canada, 2016). This language distribution is challenging for Canadian medical schools, particularly programs located in and around the province of Quebec, where physicians are often required to serve both the English and French populations. The University of Ottawa Medical Program is unique as it offers its MD program in two different streams: either in English or French. Applicants are required to choose the stream in which they wish to apply at the time of application. Although most Canadian medical programs require applicants to complete the MCAT, its utility is limited for schools in and around the province Quebec because it is only offered in English.

CASPer® is an online standardized Situational Judgment Test (SJT) that assesses the non-cognitive attributes and personal competencies of applicants such as empathy, professionalism, and communication skills. It is primarily used for predicting success in medical school. CASPer is predominantly an SJT that presents test-takers with hypothetical scenarios and asks them to describe what they would do in those situations. Unlike traditional SJTs in the multiple-choice format, CASPer is open-ended where test-takers are allowed to respond freely to the hypothetical scenarios. This approach targets respondents’ intrinsic values and beliefs by allowing them to describe not only what they would do, but why they would do it. CASPer is composed of 12 sections, eight are video-based and four are word-based. Following each hypothetical scenario, students have five minutes to respond to three probing questions. The total test time is approximately 75 minutes.

Previous studies have demonstrated the strong psychometric properties of CASPer. Test scores have been shown to be reliable (G-coefficient = .83 - .86; inter-rater reliability = .85 - .91; Dore et al., 2009), and predict both short-term (performance on the multiple mini-interview, Dore et al., 2009; applicant rank list position, Shippers et al., 2017) and long-term (licensure scores, Dore et al., 2017) performance in medical school. By the end of 2018, over 125,000 individuals have taken CASPer, and approximately 75% of all North American medical school applicants during the application cycle will have completed CASPer at some point during the admissions process.

CASPer is offered in both English and French, making it applicable for testing, regardless of language stream. At the University of Ottawa, candidates are required to take the CASPer test in the language stream to which they are applying. The English and French versions of CASPer are constructed and evaluated separately to account for the differing linguistic and cultural contexts of respondents ( Laroche et al., 1996). While cultural context is important to take into account, it is just as important to establish that each version of the test has comparable psychometric properties. This ensures symmetry and fairness in the admissions process for all applicants. In sum, both the English and French versions of CASPer should effectively assess personal competencies necessary for becoming a successful physician. This also requires that both versions are equally difficult, so applicants are not advantaged or disadvantaged by applying to one language stream over another.

The aim of the present study is to compare the characteristics of the English and French version of CASPer to examine their psychometric equivalency. Specifically, we sought to determine whether the English and French versions of CASPer demonstrate equal internal consistency and equal difficulty. We further examined potential differences in test-taking behaviour between English and French applicants by comparing test completion times, the number of characters typed as well as potential differences in applicant perceptions.

## Methods

This study was reviewed and approved by the Research Ethics Board (REB) of the University of Ottawa (Protocol Number 20180813-01H).

The University of Ottawa Faculty of Medicine adopted CASPer to assess applicants’ non-cognitive competencies. Because CASPer is offered on multiple dates, it requires multiple versions to deter cheating ( Cluskey Jr. et al., 2011). Scores are standardized within a single test session to control for potential differences in difficulty across test versions. However, as most of the test content does not overlap across sessions, internal-consistency reliabilities were calculated for each test session and a range of reliability scores are presented in the subsequent analyses. Additionally, because reliability scores are more precise with larger samples ( Charter, 1999), we incorporated all Canadian test-takers (n = 30,666, English = 69%, French = 31%) across all test sessions (n = 57) to compute the test reliabilities.

A total of 12,463 students completed CASPer across three years (entry 2016, entry 2017, entry 2018) for application to the University of Ottawa MD program. Of these test-takers, 1917 were repeat test-takers and 66 test-takers completed CASPer in both French and English. 92% (n = 11,441) of students completed CASPer in English, while 8% (n = 1,022) of the students completed CASPer in French. The breakdown of each cohort is presented in Table 1.

**Table 1.** Breakdown of the number of CASPer test-takers across the three cohorts at the University of Ottawa

	Entry 2016	Entry 2017	Entry 2018
English	3678	3829	3934
French	346	357	319

We assessed test difficulty by examining differences in the average means of CASPer scores between the English and French test-takers. However, as CASPer is relatively scored and the scores are standardized within a particular vertical (English, French), the means of CASPer test for the entire 2017-2018 cohort will effectively be zero with a standard deviation of one. Hence, a comparison of the means will likely reflect differences in the competitiveness of the English and French applicant pool rather than a difference in the difficulty of the test. An alternative method to assess difficulty is to compare the time taken to complete the test along with the rate of typing. These time stamps were collected most recently for the entry 2018 applicants. If students are taking a longer amount of time on the test and are typing slower, this may indicate a higher difficulty of the test.

During the 2017-2018 application cycle, an optional survey was added after completing CASPer, asking students to provide feedback about their test experience. A total of 4269 students completed the survey (English = 3,950, French = 319). The survey included the following questions in the language in which the test was taken (See Appendix A for the French version of the post-CASPer survey):

• On a scale of 1-10 (with 10 being the most positive), how satisfied are you with the ease of signing up for the CASPer test?
• On a scale of 1-10 (with 10 being the most positive), how satisfied are you with the smoothness of the CASPer test delivery?
• On a scale of 1-10 (with 10 being the most positive), how satisfied are you with the overall CASPer test experience?
• On a scale of 1-10 (with 10 being the most positive), how well do you think the CASPer test will differentiate your personal characteristics compared to other applicants?
• On a scale of 1-10 (with 10 being the most positive), does having the CASPer test as a requirement make you more likely or less likely to apply to a school?

## Results/Analysis

Detailed comparisons between the English and French cohorts are presented in Table 2. Due to the large sample size, even minor differences would likely be statistically significant. Therefore, we focused primarily on effect sizes to determine whether there were practical differences between the French and English groups.

**Table 2.** Comparison of CASPer properties between English and French test-takers

	English	French
Mean	0.056 n = 11,366	-0.052 n = 1,009
SD	0.989 n = 11,366	1.008 n = 1,009
Internal-consistency reliability (alpha)	0.78 (mean) 0.70 - 0.91 (range) n varies depending on test session	0.77 (mean) 0.70 - 0.86 (range) n varies depending on test session
Time to completion ^1^	Mean = 59.7 minutes SD = 1.44 mins n = 3870	Mean = 59.8 minutes SD = 1.00 mins n = 307
Typing speed (# of characters/min)	Mean = 237 SD = 62 n = 3870	Mean = 186 SD = 54 n = 307
**Post-CASPer Survey**		
Satisfaction with sign-up	Mean = 9.15, SD = 1.36, n = 3806	Mean = 8.77, SD = 1.68, n = 314
Smoothness of delivery	Mean = 9.12, SD = 1.39, n = 3801	Mean = 8.40, SD = 1.82, N = 315
Overall test experience	Mean = 8.21, SD = 1.80, n = 3802	Mean = 7.86, SD = 1.86, n = 315
Face validity	Mean = 6.18, SD = 2.12, n = 3802	Mean = 6.55, SD = 2.46, n = 313
Likelihood of applying to program requiring CASPer	Mean = 5.64, SD = 2.29, N = 3805	Mean = 7.52, SD = 2.51, N = 312

^1^ Disregarding the optional 15‐minute break after the completion of 6 stations, the maximum amount of time is 60minutes.

### Test Difficulty

**Figure 1.**
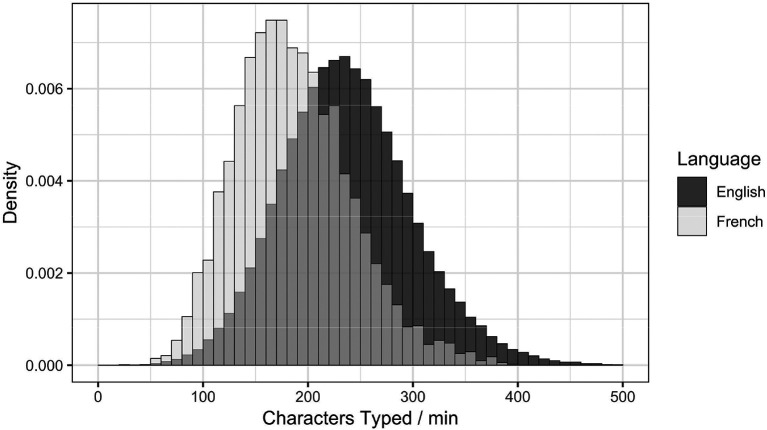
A comparison of typing speed between English and French test-takers

The English test-takers scored approximately .11 of a point higher than the French test-takers. Despite both groups taking the full hour to complete CASPer, on average, French test-takers typed more slowly than English test-takers [See Figure 1 for typing speed distributions]. However, it is unclear whether this discrepancy is due to the French version of the test being more difficult, French applicants being less competitive from the non-cognitive attributes perspective, some French test-takers being less comfortable with the French language, or linguistic differences between French and English.

### Psychometric characteristics

The scores from both groups were normally distributed. Despite minor differences in test-taking behaviour described previously, the large overlap in CASPer scores indicates that the difference between groups was small. The internal-consistency reliabilities of CASPer were the same for both groups, with all reliabilities greater than .7. This suggests that the English and French versions of CASPer are equally reliable.

**Figure 2.**
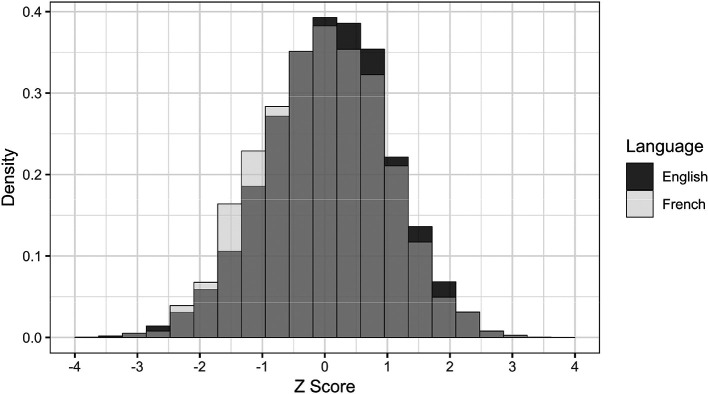
Distribution of CASPer scores between English and French test-takers

### Applicant perceptions

Although both groups had a positive experience with CASPer, French test-takers’ experience was slightly less positive. They were less satisfied with the sign-up process (diff = 0.38, t(4118) = 4.67, p < .001), smoothness of delivery (diff = 0.72, t(4114) = 8.60, p < .001), and overall test experience (diff = 0.35, t(4115) = 3.30, p < .001). In spite of this, French test-takers thought CASPer had greater face validity (diff = -0.37, t(4113) = 2.93, p < .01), and were more likely to apply to schools requiring it than English test-takers (diff = -1.89, t(4115) = 13.84, p < .001). Surprisingly, French test-takers’ less positive experience did not negatively affect how valid and useful they thought of CASPer.

## Discussion

Overall, English and French applicants exhibited slightly different test-taking behaviours. French applicants typed more slowly than English applicants. This could have been because the French version of the test was more difficult. It also could have been due to cohort differences. Some French applicants to the University of Ottawa MD Program may be slightly less comfortable test-taking in French compared to English applicants testing in English, reflected in the discrepancy between each group’s average CASPer score. Despite these small cohort differences, this had no impact on the likelihood of being accepted to either stream as the two different cohorts applying to the University of Ottawa are ranked separately (within cohorts only). Regardless of why small differences occur between English and French test-takers, we were not able to compare the scores directly. Therefore, it is advisable that bilingual English-French programs adopting CASPer assess applicants to each language stream separately.

Nevertheless, the small differences in test-taking behaviour did not impact CASPer’s reliability or face validity. Scores for both the English and French versions were highly reliable, and both groups thought the test was a valid indicator of personal characteristics. French test-takers’ slightly less positive experience may be because there is little information about CASPer available in French. To remedy this issue, more effort should be made to provide French applicants with information about CASPer. Despite having a less positive experience, French test-takers found CASPer to have a slightly face validity than the English test-takers. These results provide preliminary evidence that the strong psychometric properties of the English version also extend to the French version of this test.

## Conclusion

Determining that CASPer retains its psychometric properties when translated into different languages increases its widespread applicability. In addition to the French-English bilingual medical schools in Canada, other countries also offer medical education in more than one language. For instance, China Medical University and Shanghai Jiao Tong University School of Medicine teach in English and Mandarin. There are also a number of institutions that incorporate both English and Spanish, including The University of Texas Medical Branch, The University of Arizona College of Medicine, and Universidad Autónoma de Guadalajara. The ability to translate CASPer into different languages and still maintain its reliability and validity makes it an invaluable addition to the admissions process.

## Take Home Messages

• Medical admissions can be particularly challenging for programs which accept applicants in different languages, as they need to ensure that the tools they use in the admissions process are psychometrically equivalent across the different languages.
• CASPer is a non-cognitive admissions test that is offered in both French and English to meet the needs of the bilingual community in Canada.
• A comparison between the English and French versions demonstrate potentially small differences in test difficulty, which did not impact the psychometric properties of CASPer lending support to its use in both communities.

## Notes On Contributors

Christopher Zou, Ph.D. is Research Scientist at Altus Assessments.

Meghan McConnell, Ph.D. is Department of Innovation in Medical Education and Assistant Professor at University of Ottawa.

John J. Leddy, Ph.D. is Director of Evaluations for the MD program and Associate Professor at University of Ottawa.

Patrick Antonacci, M.Sc. is Data Scientist at Altus Assessments.

Geneviève Lemay, M.D. is Assistant Dean, Admissions for Faculty of Medicine and Assistant Professor at University of Ottawa.

## Appendices

Appendix A

French version of the Post-CASPer Survey

• Sur une échelle de 1 à 10 (10 étant le plus positif ), veuillez préciser votre niveau de satisfaction concernant le processus d’inscription à l’examen CASPer
• Sur une échelle de 1 à 10 (10 étant le plus positif ), veuillez préciser votre niveau de satisfaction concernant le déroulement de l’examen CASPer
• Sur une échelle de 1 à 10 (10 étant le plus positif ), veuillez préciser votre niveau de satisfaction concernant l’ensemble de votre expérience CASPer
• Sur une échelle de 1 à 10 (10 étant le plus positif ), veuillez préciser si vous pensez que l’examen CASPer vous permettra de vous démarquer des autres candidats en ce qui concerne vos compétences transversales
• Sur une échelle de 1 à 10 (10 étant le plus probable), veuillez préciser si vous feriez une demande d’admission auprès d’un autre programme dans le cas où CASPer est utilisé dans le cadre du processus de sélection

## Declarations

Christopher Zou and Patrick Antonacci are employed by Altus Assessments, the company responsible for the construction and delivery of the CASPer test.

## References

[ref1] Charter R.A. (1999) Sample size requirements for precise estimates of reliability, generalizability, and validity coefficients. Journal of Clinical and Experimental Neuropsychology. 21(4), pp.559–566. 10.1076/jcen.21.4.559.889 10550813

[ref2] Cluskey G.R. Jr. Ehlen C.R. and Raiborn M.H. (2011) Thwarting online exam cheating without proctor supervision. Journal of Academic and Business Ethics. 4(1).

[ref3] Dore K.L. Reiter H.I. Eva K.W. Krueger S. (2009) Extending the interview to all medical school candidates-Computer-Based Multiple Sample Evaluation of Noncognitive Skills (CMSENS). Academic Medicine. 84(10), pp.S9–S12. 10.1097/ACM.0b013e3181b3705a 19907396

[ref4] Dore K.L. Reiter H.I. Kreuger S. and Norman G.R. (2017) CASPer, an online pre-interview screen for personal/professional characteristics: prediction of national licensure scores. Advances in Health Sciences Education. 22(2), pp.327–336. 10.1007/s10459-016-9739-9 27873137

[ref5] Laroche M. Kim C. Hui M.K. and Joy A. (1996) An empirical study of multidimensional ethnic change: The case of the French Canadians in Quebec. Journal of Cross-Cultural Psychology. 27(1), pp.114–131. 10.1177/0022022196271008

[ref6] Shipper E.S. Mazer L.M. Merrell S.B. Lin D.T. Lau J.N. (2017) Pilot evaluation of the Computer-Based Assessment for Sampling Personal Characteristics test. Journal of Surgical Research. 215, pp.211–218. 10.1016/j.jss.2017.03.054 28688650

[ref7] Statistics Canada . (2017) English, French, and Official Language Minorities in Canada. Ottawa: Statistics Canada Catalogue no. 98-200-x. Available at: https://www12.statcan.gc.ca/census-recensement/2016/as-sa/98-200-x/2016011/98-200-x2016011-eng.cfm

