# Supplementary material for: Comparison of the English and French versions of the CASPer® Test in a bilingual population
Source: MedEdPublish (2016). 2018 Dec 11;7:281. [Version 1] doi: 10.15694/mep.2018.0000281.1 (PMC10711994; doi:10.15694/mep.2018.0000281.1)
Supplement: Peer review history

# Reviewer report: Reviewer response for version 1

**DOI:** 10.21956/mep.19729.r29551

**Published:** 2019-01-04

**Authors:** Hassaan Waqar

**Affiliations:** 1 St Helens and Knowsley Teaching Hospitals NHS Trust – Lead Employer

This review has been migrated. The reviewer awarded 4 stars out of 5 A very interesting and relevant article regarding the use of a bilingual admission tool for a bilingual medical program in Canada. The abstract is an accurate summary of information contained in the article. The introduction adequately introduces the problem of using a fair and valid tool for admission to the two language streams. However, from a layout point of view perhaps the mention of other bilingual programs may have been more appropriate in the introduction rather than later in the conclusion. The methods section describes the process of carrying out the project, however typing speed and length of time taken to complete the test are variables that could have been affected by a number of different factors other than difficulty, which could have influenced the results. Whilst this is addressed in the results section, it may have been useful to discuss the results of the students who took both the French and English versions of the selection tool. Whilst this cohort was small, it may have revealed interesting results which could have added an extra facet and depth to the discussion. The discussion explores implications of the results of the study to a reasonable depth. The conclusion makes reference to the admission tool retaining its reliability and validity when used in different languages, which may imply languages other than French and English. However, such a conclusion without reliability and validity data on languages other than French and English may not be justified. Further studies may be needed in order to determine if such a conclusion is appropriate.

Reviewer Expertise:

NA

No decision status is available

# Reviewer report: Reviewer response for version 1

**DOI:** 10.21956/mep.19729.r29552

**Published:** 2018-12-14

**Authors:** Richard Hays

**Affiliations:** 1 James Cook University

This review has been migrated. The reviewer awarded 4 stars out of 5 Many thanks for the invitation to review this interesting paper. Whatever one thinks about individual selection tools and methods (I still have some concerns about SJTs for 17 year olds), it is essential that we address linguistic and cultural bias if the tests are to be administered to different language/cultural groups. Simple translation does not work so essentially different tests can result. Canada perhaps does this better than most places, particularly in assessment. I agree that the results demonstrate that the two versions of the test seem about equal and to be appropriate for their different populations. As ranking will occur within the two groups in any case, the differences are relatively minor and unlikely to affect the quality of candidates selected. My only concern is the method of measuring test difficulty. I am not sure that typing speed is That meaningful. Ultimately, longer term follow up should show if there are any differences in postgraduate career choice and success, but I suspect nothing will show up there, as there are so many influences between selection into medicine and final careers.

Reviewer Expertise:

NA

No decision status is available

# Reviewer report: Reviewer response for version 1

**DOI:** 10.21956/mep.19729.r29553

**Published:** 2018-12-11

**Authors:** Balakrishnan Nair

**Affiliations:** 1 Centre for Medical Professional Development and University of Newcastle

This review has been migrated. The reviewer awarded 4 stars out of 5 Medical school selection is a challenging issue. More and more schools are doing non-cognitive testing to pick up the right students for training. Language skills could be an impediment to this form of testing. This interesting study which compared the English and French version of the CASPer test and concluded that there is no difference. Both versions retained the psychometric properties. The methodology and sample size are adequate, and the conclusions are valid

Reviewer Expertise:

NA

No decision status is available

# Reviewer report: Reviewer response for version 1

**DOI:** 10.21956/mep.19729.r29550

**Published:** 2018-12-11

**Authors:** Virginia Randall

**Affiliations:** 1 Uniformed Services University of the Health Sciences

This review has been migrated. The reviewer awarded 4 stars out of 5 Thanks to the authors for tackling a vexing problem for bilingual assessment for bilingual medical schools. Surely the good results are due to the similarity of cultural approaches to medical care in the two linguistic populations of Quebec. The translation and "grading" by the standardized assessors is a remarkable and tedious task which was obviously done well in this situation.

Reviewer Expertise:

NA

No decision status is available
